# Recovery of non-reference sequences missing from the human reference genome

**DOI:** 10.1186/s12864-019-6107-1

**Published:** 2019-10-16

**Authors:** Ran Li, Xiaomeng Tian, Peng Yang, Yingzhi Fan, Ming Li, Hongxiang Zheng, Xihong Wang, Yu Jiang

**Affiliations:** 1 Key Laboratory of Animal Genetics, Breeding and Reproduction of Shaanxi Province, College of Animal Science and Technology, Northwest A&F University, Yangling, 712100 China; 2 Human Phenome Institute, Fudan University, Shanghai, 200438 China

**Keywords:** Pan-genome, Human genome, Genomic variation, Alternate alleles

## Abstract

**Background:**

The non-reference sequences (NRS) represent structure variations in human genome with potential functional significance. However, besides the known insertions, it is currently unknown whether other types of structure variations with NRS exist.

**Results:**

Here, we compared 31 human de novo assemblies with the current reference genome to identify the NRS and their location. We resolved the precise location of 6113 NRS adding up to 12.8 Mb. Besides 1571 insertions, we detected 3041 alternate alleles, which were defined as having less than 90% (or none) identity with the reference alleles. These alternate alleles overlapped with 1143 protein-coding genes including a putative novel MHC haplotype. Further, we demonstrated that the alternate alleles and their flanking regions had high content of tandem repeats, indicating that their origin was associated with tandem repeats.

**Conclusions:**

Our study detected a large number of NRS including many alternate alleles which are previously uncharacterized. We suggested that the origin of alternate alleles was associated with tandem repeats. Our results enriched the spectrum of genetic variations in human genome.

## Background

The initial human reference genome was entirely linear [1]. Despite introduction of alternate alleles for a graph-based representation, the current reference genome is largely derived from a single individual of African-European origin [2], limiting its representation of diverse populations. Lines of evidence in recent years have revealed that individuals still carry sequences that are not represented in the reference genome. These sequences could be an important type of structural variation underlying disease associations or complex traits [3]. The discovery of non-reference sequences (NRS) will be a prerequisite for an more complete graph-based genome, thereby enabling improved genomic analyses and understanding of genomic architecture [4].

Extensive efforts have been devoted in recent years to discover NRS. Based on a large amount of whole-genome sequencing data, two studies focused specifically on the discovery of NRS and identified as much as ~ 300 Mb novel sequences from a large number of re-sequencing data [3, 5]. However, the identified sequences were obtained by assembling unaligned short reads, posing a challenge to placing them in the reference genome. A recent study used long-read sequencing data from 15 samples to identify 32,838 insertions of presence in at least two samples but without exploring novel sequences within the insertions [6]. Additionally, these studies have mainly focused on insertion events. In fact, some sequences belong to complex structural variants (e.g., two alleles with high divergence instead of simply introducing additional sequences) [5, 7]. De novo assembly is a promising approach for building the complete human pan-genome [7]. Using an assembly-versus-assembly approach, we discovered not only insertions but also sequences which are an alternate representation of a locus in the haploid genome. A well characterization of the insertions and alternate alleles in the human genome is necessary for a better understanding of their biological significance.

The identification of insertions and alternate alleles requires high-quality de novo assemblies. Fortunately, a number of human de novo assemblies have been generated via long-read sequencing (LRS) [6, 8–12], and these assemblies have covered major human ethnic groups. The unprecedented availability of these genomic resources enables us to depict the full spectrum of NRS, especially those representing alternate alleles. In this study, we compared 31 de novo assemblies (including 17 LRS assemblies) with the human reference genome to identify putative alternate alleles, most of which are newly reported in this study.

## Results

### Detection of candidate NRS

**Fig. 1:**
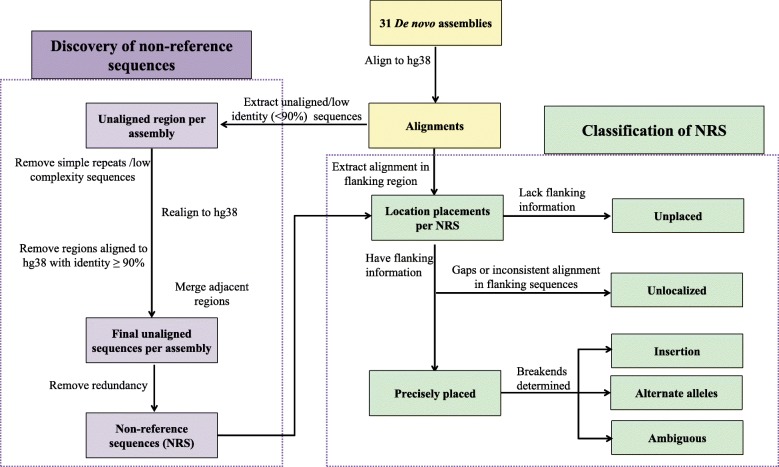
Overview of the workflow to identify non-reference sequences (NRS)

An assembly-versus-assembly approach was used for each assembly to identify unaligned sequences (< 90% identity) to the human reference genome (GRCh38.p12, hg38) (Fig. 1, Methods). Apart from hg38, our study included 31 de novo assemblies: 17 from LRS (PacBio or nanopore sequencing technology), 13 generated with next generation sequencing and one from Sanger sequencing (Additional file 1). We further examined the BUSCO completeness score, length distribution of structural variants as assessed by Assemblytics and composition of TE elements to ensure that these assemblies are of high quality for downstream analysis. All the assemblies present comparable completeness scores (94.4 ± 0.81, mean ± SD) except for ASM101398v1 (BUSCO score 90.3) and the percent of TE elements in all assemblies were all at similar levels (0.51 ± 0.01, mean ± SD) (Additional file 1). All the assemblies shows similar pattern of length distributions of structural variants (Additional file 2).

**Fig. 2:**
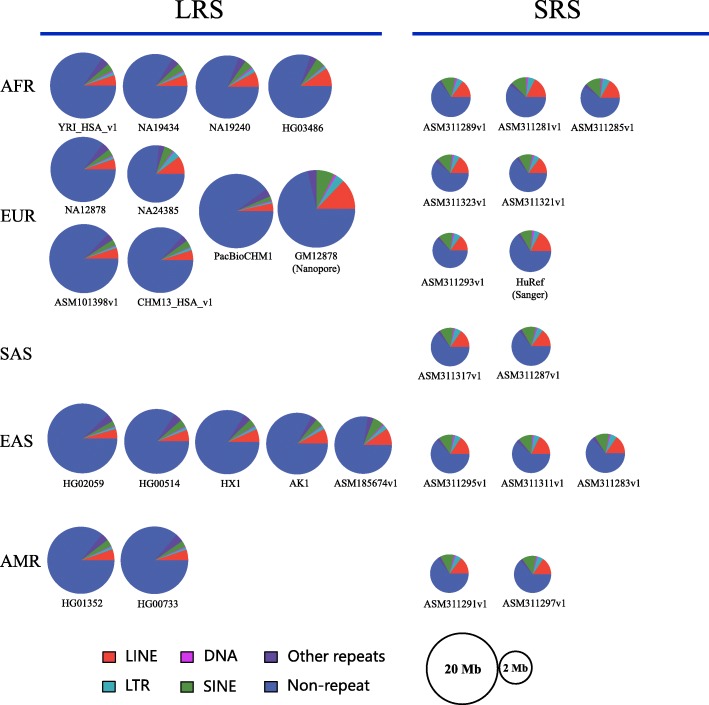
Non-reference sequences identified from each of the 31 human de novo assemblies. The repeat information was summarized from the repeat annotation files (*_rm.out.gz), which were generated with RepeatMasker and downloaded from the NCBI. The radius of each pie chart was log2 transformed

The final unaligned sequences spanned, on average, 12.9 Mb for LRS assemblies, which were much larger than the other assemblies (2.3 Mb on average) (*P* < 0.01, unpaired two-tailed Student’s t-test) (Fig. 2). For each assembly, 70-80% of the preliminary unaligned sequence contents belonged to simple sequence repeats or low complexity sequences and were removed to obtain the final unaligned sequences for each assembly. After removing redundant sequences and sequences shorter than 400 bp, we obtained the unaligned sequences from each assembly, which were then merged into a unified non-reference call set of 15,055 sequences (hereafter referred to as NRS) adding up to 129.1 Mb with a median length of 2848 bp (N50 = 1066 bp). Furthermore, 78.6 Mb of the NRS had no alignment with hg38 using the criteria of > 80% identity and 50% coverage as adopted by [5]. Nevertheless, the call set of 129.1 Mb were used in downstream analysis. In order to compare with four previous studies (methods), we aligned our NRS to each of the four datasets in a reciprocal manner with BLAST to determine the intersection with each of the studies. In total, 13.8 Mb NRS intersected with previous reports whereas the rest (115.3 Mb) has not been identified before (Additional file 3).

We aligned all the NRS to genomes of four great apes and found that 1211 sequences (4.32 Mb) were present in at least one of the four great apes (identity ≥95% and coverage ≥80%). The presence of each NRS was also examined in each of the human de novo assemblies and those which were present in at least two of the 31 de novo assemblies and great ape assemblies were determined as non-private sequences. Taken together, 28.2% (4248) of the NRS spanning 29.3 Mb were non-private sequences, indicating that they are of high confidence (Methods and Additional file 4).

### Placing candidate NRS to the reference genome

We next determined the genomic locations of the NRS by aligning their flanking sequences to hg38 (Methods), by which we resolved the precise location of 6112 NRS (40.5% of the total NRS) adding up to 12.8 Mb. Notably, 13 sequences reside in the remaining gaps of the reference genome (Additional file 5). Another 2711 NRS were anchored to chromosomes without a precise location due to sequence gaps. The remaining sequences cannot be placed on hg38 due to a lack of flanking sequences or conflict anchoring information from the two sides.

For the precisely placed sequences, we can determine whether they belong to insertions or alternate alleles as described in next section. For the unplaced NRS and those without precise locations, 2855 were non-private sequences adding up to 25.8 Mb, indicating that they are of high confidence (Additional file 6). Although we could not determine the precise locations of these sequences, their discovery will greatly expand the repertoire of sequence diversity in the human genome.

### Insertions within NRS

**Fig. 3:**
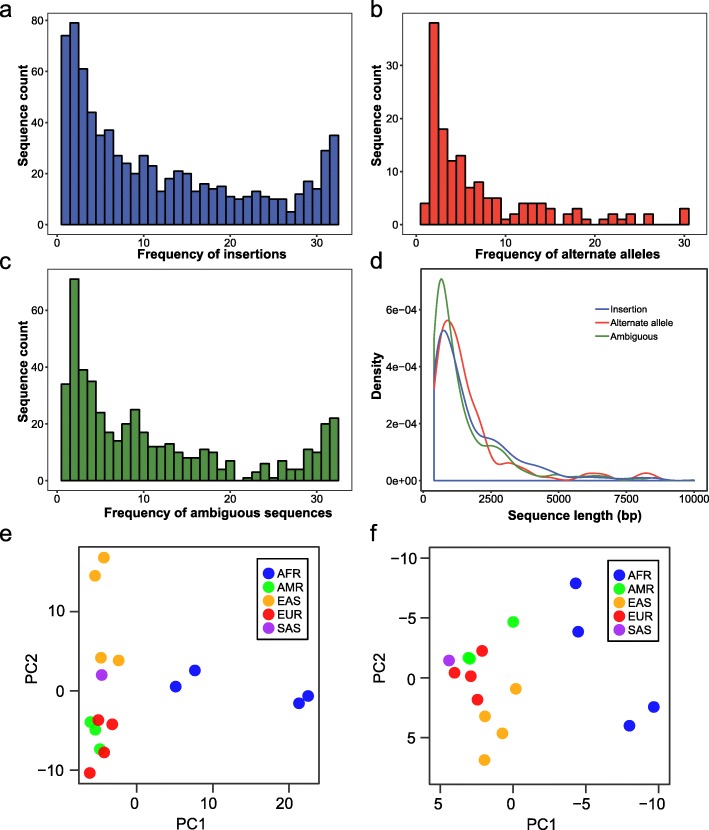
Characteristics of the insertions and alternate alleles. **a** Frequency of insertions within the 31 de novo assemblies. **b** Frequency of alternate alleles within the 31 de novo assemblies. **c** Frequency of ambiguous sequences within the 31 de novo assemblies. **d** Length distributions of the insertions, alternate alleles and ambiguous sequences. **e** The first two principal components based on the occurrence matric of the insertions among the 16 LRS de novo assemblies. **f** The first two principal components based on the occurrence matric of the alternate alleles among the LRS de novo assemblies

We first determined the insertion events for the precisely placed NRS. A total of 1571 (3.2 Mb) were found to be insertions including 769 non-private sequences (Fig. 3a). Furthermore, 246 were present in more than half of the assemblies, indicating that they could represent major alleles. Notably, 56.8% (881) of the insertions, including 158 non-private ones, were firstly described in our study. Principal component analysis (PCA) of all the insertions based on their presence in the 16 LRS de novo assemblies of Pacbio sequencing showed a population-specific pattern (Fig. 3e). PC1 clusters African samples away from other populations, while PC2 further separates the East Asians from the Europeans, Americans and South Asians.

### Alternate alleles within NRS

**Fig. 4:**
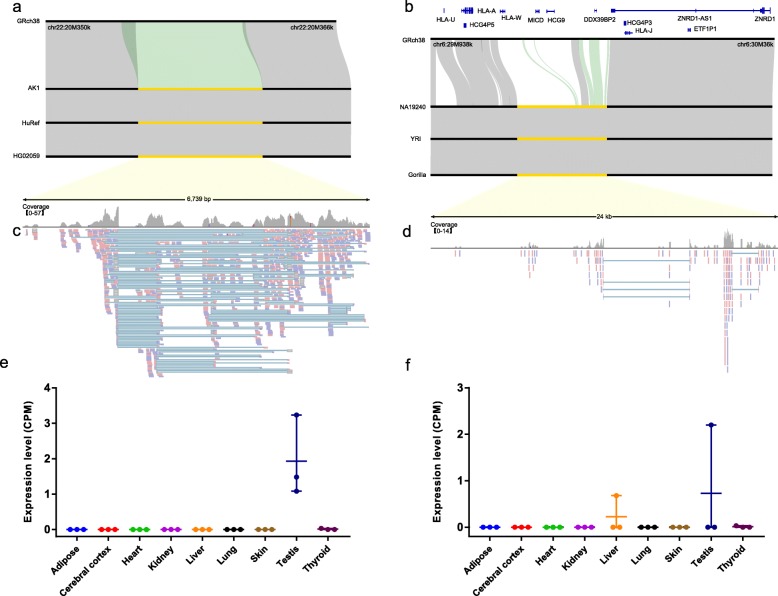
Examples of alternate alleles. **a** and **b** Alignment of the alternate alleles and their flanking sequences with hg38 and other assemblies. The blue lines in the top represent the gene annotations in hg38. The yellow segments represent the NRS. The gray block represents alignments that share ≥95% identity, while the green block represents alignments that share < 90% identity. For each of the alignments, the reference sequence from hg38 is shown at the top following by the sequence where the NRS is derived from and sequences from two additional genomes. **c** and **d** RNA-seq reads mapping of the NRS shows expression potential. **e** and **f** Expression of the alternate alleles in nine tissues. Three replicates were used for each tissue. The expression level was measured using CPM (reads count per million of total mapped reads)

We further found that many NRS represent an alternate allele to their counterparts in the reference instead of insertions. To identify alternate alleles, the NRS should share less than 90% identity (or none) and have comparable length with reference alleles (Methods). In this way, 3041 NRS (6.38 Mb) were identified as candidate alternate alleles. The remaining 1500 precisely placed NRS did not meet our criteria of insertions or alternate alleles and thus were classified as ambiguous sequences. Unlike insertions, the alternate alleles represent allelic sequences (Fig. 4a and d). Notably, a long alternate sequence of 24,676 bp was anchored to chr6: 29,955,749-29,986,299, which belongs to the class I major histocompatibility complex (MHC gene) (Fig. 4b) and potentially harbors a novel HLA-B gene (Additional file 7). This allele was present in two African assemblies (YRI, NA19240), in one American assembly (ASM311317v1) and in the gorilla genome whereas absent in other assemblies. Moreover, the reference allele was found in chimpanzees, indicating the presence of ancestral polymorphism at this locus. Furthermore, this alternate allelic sequence was not reported in the NCBI nr/nt database or in the human HLA database (IPD-IMGT/HLA database), suggesting that this alternate sequence represents a putative novel MHC allele.

**Fig. 5:**
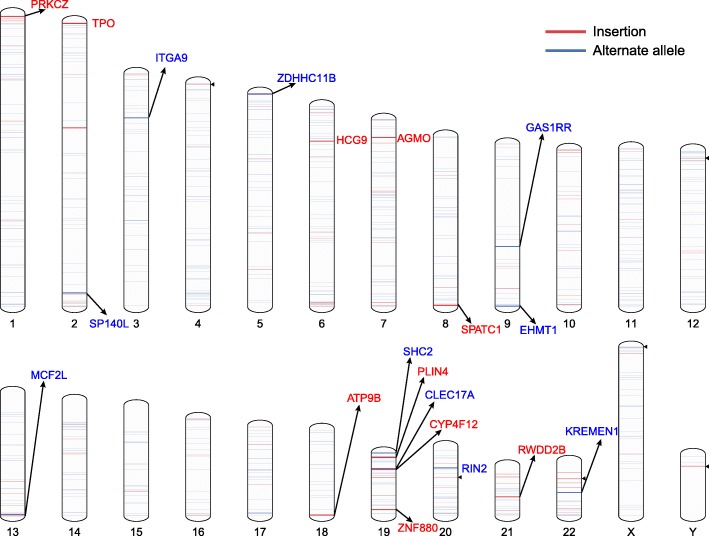
Locations of the non-private insertions and alternate alleles on the human reference genome (hg38). Red lines represent insertions, while blue lines represent alternate alleles. The gene symbol is shown for each of the ten longest insertions and ten longest alternate alleles overlapping genic regions. The black triangles represent the NRS overlapping with the gap regions. Line width is not drawn to scale

Among the alternate alleles, 1348 intersected with the genic region of 1143 protein-coding genes. The genomic distribution of the alternate alleles was dispersed throughout the genome, and those belonging to non-private sequences are shown in Fig. 5. A total of 59 non-private alternate alleles intersected with the genic region, and five of them further intersected with the CDS region of eight genes: HLA-W, MICD, HCG9, DDX39BP2, LOC107985837, ZNF880, PLIN4 and LOC728715.

Only 2.6% (80 out of 3041) of the alternate alleles have been identified before but were miss-classified as insertions. Therefore, most of the identified alternate alleles described in our study are newly reported. The discovery frequency of alternate alleles in human assemblies appears to be lower than that of insertions (Fig. 3a and b). Most alternate alleles were present in less than half of the 31 assemblies, indicating that many of them represent minor alleles in the human genome. Nevertheless, 144 alternate alleles were non-private (Additional file 8), with the longest one found in 17 assemblies and spanning 19,330 bp (genomic placement position: chr7, 62,408,641-62,451,864). The ambiguous sequences also included a number of putative insertions and alternate alleles (Fig. 3c) and deserve further efforts for verification. The length distributions of the alternate alleles and insertions did not differ (*p* = 0.09, Kolmogorov-Smirnow test) (Fig. 3d).

Similar to the insertions, PCA also showed that PC1 clusters African samples away from other populations, while PC2 separates the East Asians from the Europeans, South Asians and Americans (Fig. 3f). We also explored the potentially transcribed sequences that either have mapped RNA-seq reads (≥10 reads in at least two samples) or hits to the human expressed sequence tag database (dbEST) (e-value <1e-5). We totally identified 74 transcribed alternate alleles from RNA-seq data and 238 with hits to the human dbEST, resulting in a total of 244 potentially transcribed sequences (Additional file 9). One alternate allele was found to be expressed in a tissue-specific manner (Fig. 4b and c), which is potentially a long non-coding gene since we couldn’t annotate it to any known gene by searching NCBI nr/nt database with BLAST. The putative novel MHC allele was also found to be expressed (Fig. 4e and f).

**Fig. 6:**
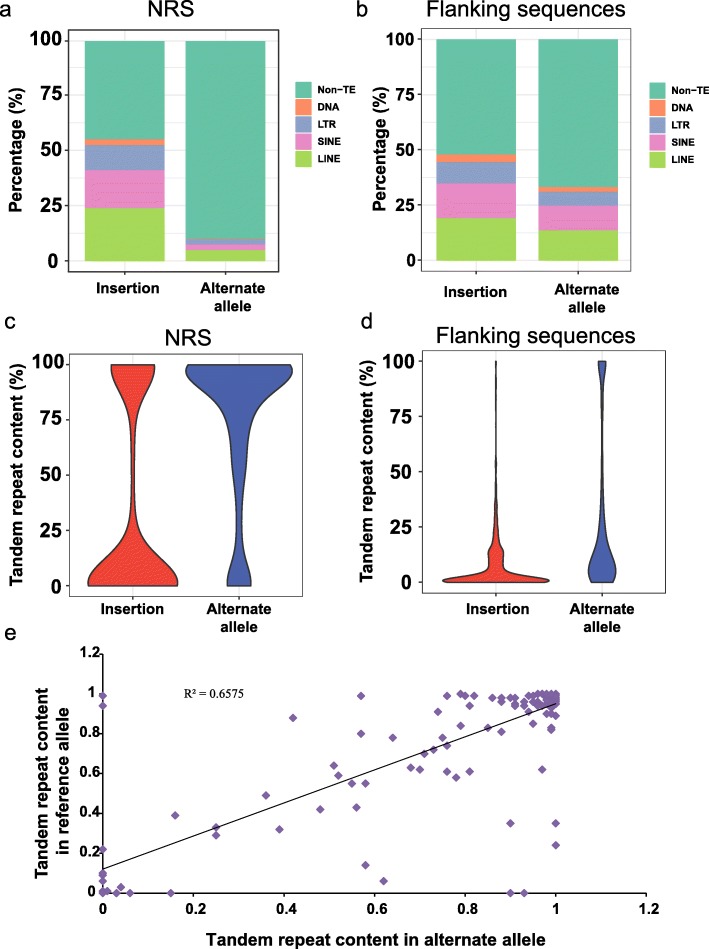
The repeat contents associated with the insertions and alternate alleles. **a** TE content within the insertions and alternate alleles; **b** TE content in the flanking region (5 kb on each side). **c** The tandem repeat content within the insertions and alternate alleles. **d** The tandem repeat content in the flanking region (5 kb on each side). The non-private sequences were included for statistics. **e** Dot plot showing the tandem repeat content in alternate allele (x axis) and in the corresponding reference allele

To explore the origin of the alternate alleles, we analyzed associated repeats with NRS. Transposable elements (TEs) compose approximately 45% of the human genome (Lander et al. 2001), and a previous study showed that insertions were associated with TEs [13]. The percent of TEs within alternate alleles (10.0%) was much lower than that of insertions (55.1%) (Fig. 6a). The flanking sequences (5 kb on each side) of the alternate alleles also had less TE content (33.3%) than the insertions (48.0%) (Fig. 6b), suggesting that the alternate alleles are not associated with TEs. We then screened the tandem repeat content among the sequences. The alternate alleles possessed a much higher content of tandem repeats either within the sequences (Fig. 6c) or in the flanking sequences (5 kb on each side, Fig. 6d) compared with the insertions. Notably, the reference allele also be enriched in tandem repeat when the alternate allele have a large content of tandem repeat (*R*^2^ = 0.65, Fig. 6e and Additional file 10), thereby implying that tandem repeats are associated with alternate alleles.

## Discussion

A comprehensive characterization of structural variations is essential for studies attempting to identify causative variants that affect phenotypic variations and complex genetic diseases. In this study, we identified 129.1 Mb candidate NRS (4.2% of the genome). Although many of the NRS were singletons, a considerable number of reliable NRS (29.3 Mb) were identified by their presence in at least two assemblies, and most of them were newly described. The discovery of these NRS will contribute to a final, comprehensive pan-genome capturing all of the DNA present in humans.

We compared our results with four of the recent studies and only 13.8 Mb (10.7%) NRS were found to be previously reported. The discrepancy could be probably explained by the complexity in data types/size and analysis methodology. Compared with short-reads sequencing data, long-reads sequencing is capable of capturing complex genomic regions such as high/low GC content, tandem repeats, and interspersed repeat regions by generating long-reads [14], thereby enabling the discovery of more repeats or lineage-specific expansions. On the other hand, if the datasets is large enough, many NRS including some complex regions could still be found even using short-reads data as conducted in the African pan-genome (910 genomes) [5]. These above mentioned factors could also partially explain why many of the NRS were not found in primates. Actually, we found that only 4.1 Mb (1.38%) of the African pan-genome can align to the primates’ genomes using BLAST. Furthermore, even the definition of non-reference sequences (or so-called novel sequences) is still blurred in pan-genomic studies, which in turn can significantly affect the amount of reported sequences. For example, the African pan-genome used an identity cutoff of 80% [5] whereas other studies choose 90% as the cutoff [7, 15, 16]. Since the human and chimpanzee share genome-wide nucleotide identity of ~ 98.5% despite 6 million years divergence [17], an identity of 90% is a very conservative and robust threshold for identifying human NRS.

More importantly, we discovered a large number of alternate alleles. The majority of the alternate alleles that we found have not been previously reported, which could be due to several reasons: (1) Most previous work has designed their studies to focus on insertions, whereas other types of NRS were largely ignored [3, 5, 13]; (2) Many studies have mainly relied on short-reads data to obtain NRS [3, 5], which would be less efficient for the discovery of long structural variations compared with an assembly-versus-assembly approach, as applied in our study; and (3) Many alternate alleles were singletons, suggesting that they are either of very low frequency for detection or simply false positives due to assembly errors. Nevertheless, we still detected 144 non-private alternate alleles. The current human genome (GRCh38.p12) includes 261 alternate loci that capture a limited amount of population diversity and improve read mapping for some data sets [2]. Therefore, the sequences that we identified will greatly advance our knowledge of the alternate alleles in the human genome.

There is growing interest in using genetic variants to augment the reference genome into a graph genome [18–20]. To create a representative graph genome, the full spectrum of structural variations, including the alternate alleles, should be understood clearly. With the reduction in sequencing costs and advances in sequencing technology, increased numbers of de novo assemblies will be generated, which will eventually refine the full spectrum of sequence diversities in the human genome.

## Conclusions

In this study, we identified 129.1 Mb NRS which are absent from the human reference genome, most of which have not been previously reported. For the sequences which could be precisely placed on the reference genome, we classified them into insertions and divergent alleles. Notably, 3041 alternate alleles were identified with many of them intersecting with the genic region of protein-coding genes. By examining the repeat contents within NRS and in the flanking sequences, we found that the origin of alternate alleles were probably associated with TE repeats. Our results indicated that abundant sequences are still missing from the human reference genome despite great advances in recent years and more genomic data from diverse populations are demanded to build the complete human pan-genome. Meanwhile, the biological significance of the NRS needs to be further explored.

## Methods

### De novo assemblies used in this study

The human reference genome GRCh38.p12 (hg38) was used as the guiding genome for comparison. The hg38 consists of the primary GRCh38 assembly, the mitochondria genome, unlocalized/unplaced scaffolds and alternate contigs. We downloaded 31 human de novo assemblies from the NCBI, including 17 from PacBio sequencing, 1 from nanopore sequencing, 13 from next generation sequencing and one from Sanger sequencing (Additional file 1). For the assemblies using LRS technology (PacBio and nanopore sequencing), we focused on assemblies that were released since 2015 and of high quality. All of them had high continuity (contig N50 > 1 Mb; 15 of 18 with an N50 > 5 Mb) and high sequencing coverage (16 of 17 with coverage > 50 X). For the assemblies from SRS, we used the 13 haploid genomes of next generation sequencing since they were generated in one study with high continuity [13]. The HuRef genome of Sanger sequencing was also included in our study due to its high continuity [21]. The BUSCO completeness score for each assembly was determined using BUSCO v3 [22]. The structural variations were assessed using online tool Assemblytics [23] by providing the Mummer v3.23 alignment output in delta format (nucmer -maxmatch -l 100 -c 500) [24].

### Recovery of candidate NRS from each assembly

Each assembly was aligned to hg38 using LAST (−m100 -E0.05) [25]. The unaligned or low-identity sequences (< 90%) to hg38 with a length of at least 100 bp were extracted. The unaligned or low-identity sequences identified by LAST were then aligned back to hg38 using BLAST v2.2.31 (megablast) [26] to further remove regions that share ≥90% identity. Then, the simple repeats, low complexity regions and microsatellites were removed based on the repeat annotation file, which was downloaded from the NCBI (*_rm.out.gz). The remaining sequences were merged by adjacent regions (≤200 bp), and the resulting NRS, which were at least 400 bp, were retained for each assembly. Finally, the resulting sequences were aligned to hg38 using BLAST (megablast) again to remove the regions that share ≥90% identity. The resulting sequences were then merged by adjacent regions (≤200 bp), and only those of at least 400 bp were kept. BEDTools v2.25.0 was used in the above processes when assessing genomic features [27]. The NRS from all 31 assemblies were then merged to remove redundancy and to generate a non-redundant call set using CD-HIT (−c 0.95 -aS 0.8 -d 0 -sf 1 -M 10000) [28]. The resulting unified and non-redundant call set was used for further analysis.

### Removal of contamination

We did not expect sequencing from bacteria, viruses or other non-mammalian species to be present in our identified sequences since the NCBI has a stringent quality control process when assemblies are submitted. We used BLAST (megablast) to align the non-reference call set to the NCBI nt database. Only a small number of the sequences exhibited significant alignment with the non-mammalian species using 90% identity and 90% query coverage filter thresholds and were removed from the final call set.

### Presence of NRS in de novo assemblies of 31 humans and in four great apes

We examined the presence of each NRS in 31 humans and four great ape de novo assemblies using BLAST (megablast). The four great apes included chimpanzee (GCA_002880755.3), bonobo (GCF_000258655.2), gorilla (GCA_900006655.3) and orangutan (GCF_002880775.1). The presence was determined for the NRS when having identity ≥95% and coverage ≥80% with the assembly.

### Anchoring NRS on human chromosomes

All the sequences were anchored to human chromosomes based on the LAST alignment of their flanking sequences. The anchored position was reported as ‘precisely placed’ when both of the flanking sequences were near perfectly aligned (no gap alignment) to the reference genome. If the sequences have flanking sequences of only one end aligned to the reference genome or have flanking sequences of two ends aligned but with gap alignment rendering the inference of exact breakpoints, it was reported as ‘unlocalized’. The remaining sequences were reported as ‘unplaced’. Based on the breakends coordinates (the genomic position of the two breakpoints for each NRS), the breakpoint resolved sequences could be further classified as insertions when simply introducing one sequence fragment to the reference genome. For alternate alleles, the NRS should share less than 90% (or 0%) identity with the reference allele, and the reference allele were required to be at least 400 bp. Furthermore, the NRS and the reference allele should have a comparable length, with the ratio of the length to be between 1/3 and 3. The remaining sequences that did not meet the above criteria for insertions and alternate alleles were classified as ambiguous sequences.

### Aligning NRS to the human expressed sequence tag database

We downloaded the human dbEST from the NCBI FTP site (ftp://ftp.ncbi.nlm.nih.gov/blast/db/FASTA/est_human.gz). Then, the NRS were aligned to the dbEST using BLAST. Since entries in the dbEST are short and represent only the ends of expressed genes, alignments with ≥95% identity and an e-value <1e-5 were considered as a hit regardless of the query coverage.

### Comparison with published datasets

We compared with our results with four previously published results each of which reported a list of non-reference sequences [3, 5, 13, 15]. The comparison for each of the datasets was performed using a reciprocal strategy as previously described [13]. We first aligned all the NRS to each of the datasets using BLAST. Alignments with ≥95% sequence identity and ≥ 80% query coverage were considered as real hits. We then aligned each of the four datasets to our NRS also with BLAST, and the alignments were filtered using the same criteria as described above. Results from the two alignments steps were merged and a non-redundant list of NRS was reported.

### Aligning RNA-seq reads to the primary call set

We downloaded a total of 87 RNA-seq data from the Geuvadis project (https://www.ebi.ac.uk/Tools/geuvadis-das/) and another study [29]. The information of the samples was described in Additional file 11. Fastp was used to trim off the low-quality bases (−q 20 –l 80 –u 50) [30]. Then the clean reads were mapped to the extended version of reference (hg38 plus the primary call set) using HISAT2 with default parameters [31]. Only the reads with both of the mates mapped and in proper pair were considered as high-quality alignment before counting the mapped reads on each sequence using Sambamba depth (−F “mapping_quality >= 30 and proper_pair”) [32]. A sequence was regarded to be transcribed when ≥10 mapped reads were found in at least two samples.

## Supplementary information

**Additional file 1.** Table showing the information of 31 de novo assemblies used in this study.

**Additional file 2.** The spectrum of structural variations (insertions and deletions) of the 31 de novo assemblies as assessed by Assemblytics.

**Additional file 3.** Comparison of our NRS with four published studies.

**Additional file 4.** Table showing the presence of NRS in 31 human de novo assemblies and in four great ape assemblies.

**Additional file 5.** Table showing the information of NRS which are anchored to the gap region of hg38.

**Additional file 6.** Table showing the information of the non-private NRS.

**Additional file 7. **Blastx information of the novel candidate MHC allele. **a** The two most significant hits show that the novel candidate MHC allele potentially harbors two genes; **b** Gene description of the two hits; **c** Sequence alignment of the first hit to known protein; **d** Sequence alignment of the second hit to known protein.

**Additional file 8.** Table showing the information of the precisely placed NRS.

**Additional file 9.** Table showing the list of alternate alleles with transcriptional potential.

**Additional file 10.** Two examples of the divergent alleles harboring tandem repeats.

**Additional file 11.** Table showing the list of public RNA-seq data used in this study.

**Additional file 12.** Table showing the sequence information of identified NRS.

## Data Availability

The accession numbers corresponding to each of the 31 human de novo assemblies used in our study are listed below and could be found in Additional file 1: GCA_002209525.2 (HG01352), GCA_002208065.1 (HG00733), GCA_001750385.2 (AK1), GCA_001856745.1 (ASM185674v1), GCA_002180035.3 (HG00514), GCA_003070785.1 (HG02059), GCA_001708065.2 (HX1), GCA_001524155.4 (NA19240), GCA_001542345.1 (NA24385), GCA_001297185.1 (PacBioCHM1), GCA_002077035.3 (NA12878), GCA_001013985.1 (ASM101398v1), GCA_002884485.1 (CHM13), GCA_002872155.1 (NA19434), GCA_002884475.1 (YRI), GCA_003086635.1 (HG03486), GCA_900232925.1 (GM12878), GCA_003112895.1 (ASM311289v1), GCA_003112815.1 (ASM311281v1), GCA_003112855.1 (ASM311285v1), GCA_003113175.1 (ASM311317v1), GCA_003112875.1 (ASM311287v1), GCA_003112955.1 (ASM311295v1), GCA_003113115.1 (ASM311311v1), GCA_003112835.1 (ASM311283v1), GCA_003113235.1 (ASM311323v1), GCA_003113215.1 (ASM311321v1), GCA_003112935.1 (ASM311293v1), GCA_003112915.1 (ASM311291v1), GCA_003112975.1 (ASM311297v1), and GCA_000002125.2 (HuRef). The datasets corresponding to the four great apes can be accessed on NCBI Assembly via the accession numbers: chimpanzee (GCA_002880755.3), bonobo (GCF_000258655.2), gorilla (GCA_900006655.3), orangutan (GCF_002880775.1). We downloaded the human dbEST from the NCBI FTP site (ftp://ftp.ncbi.nlm.nih.gov/blast/db/FASTA/est_human.gz). We downloaded a total of 87 RNA-seq data from the Geuvadis project (https://www.ebi.ac.uk/Tools/geuvadis-das/) and another study (Fagerberg et al., 2014). The information of the samples was described in Additional file 11. The datasets supporting the conclusions of this article are included within the article and its additional files. The identified NRS is provided as an Additional file (Additional file 12) and all other data supporting the findings of this study are available in additional files.

## Abbreviations

CDS: Coding sequence
Hg38: Genome Reference Consortium Human Build 38 (refer in particular to GRCh38.p12 in this study)
LRS: Long-read sequencing
NRS: Non-reference sequences
SRS: Short-read sequencing
TE: Transposable element
