# Supplementary figures and images for: Recovery of non-reference sequences missing from the human reference genome
Source: BMC Genomics. 2019 Oct 16;20:746. doi: 10.1186/s12864-019-6107-1 (PMC6796347; doi:10.1186/s12864-019-6107-1)

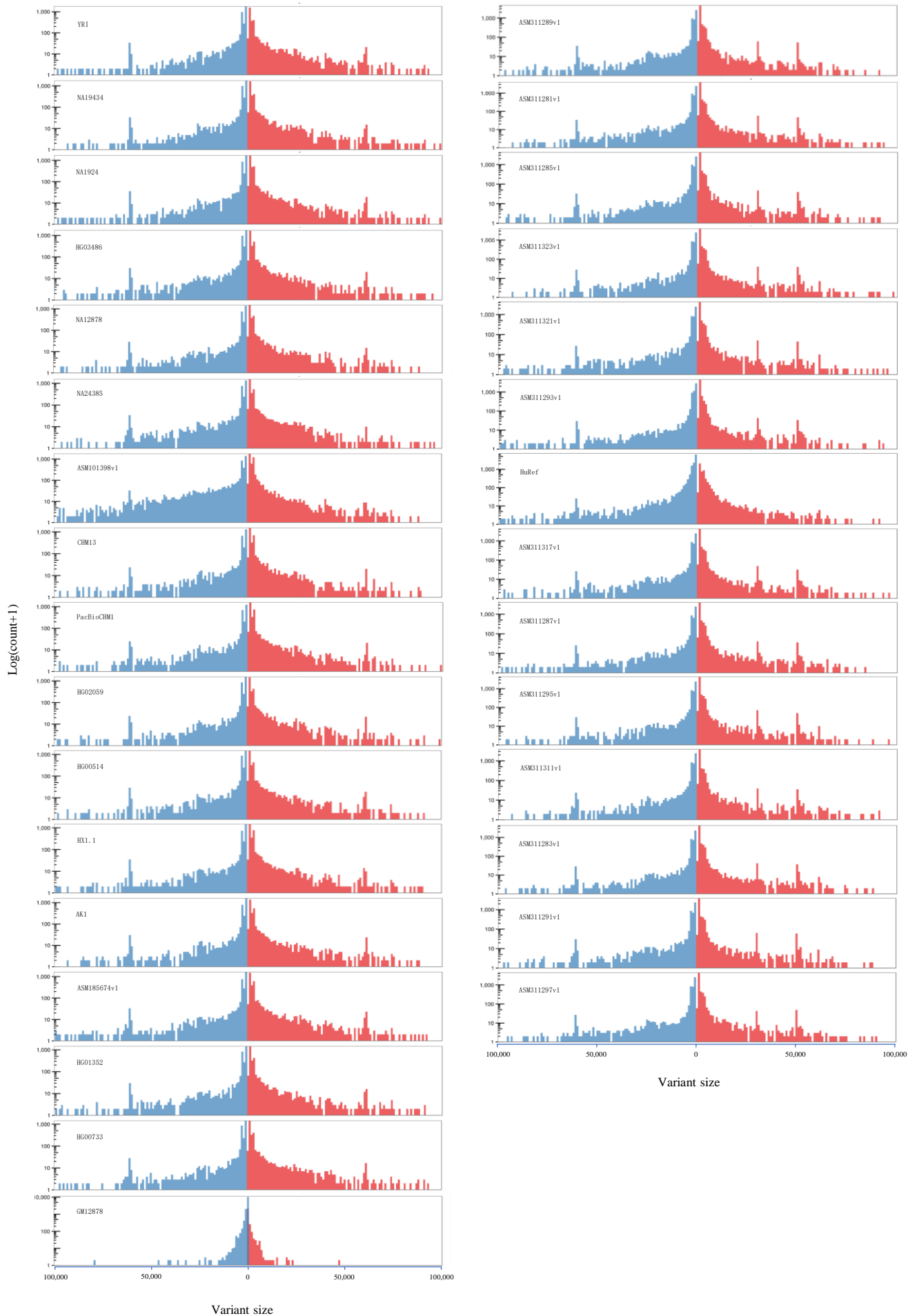

Supplement: Supplementary file 2 — Additional file 2. The spectrum of structural variations (insertions and deletions) of the 31 de novo assemblies as assessed by Assemblytics. [file 12864_2019_6107_MOESM2_ESM.pdf]

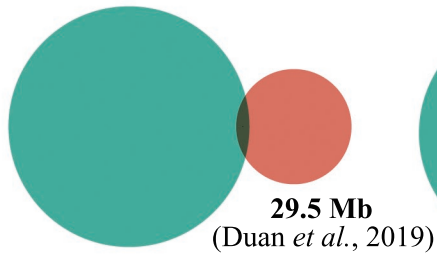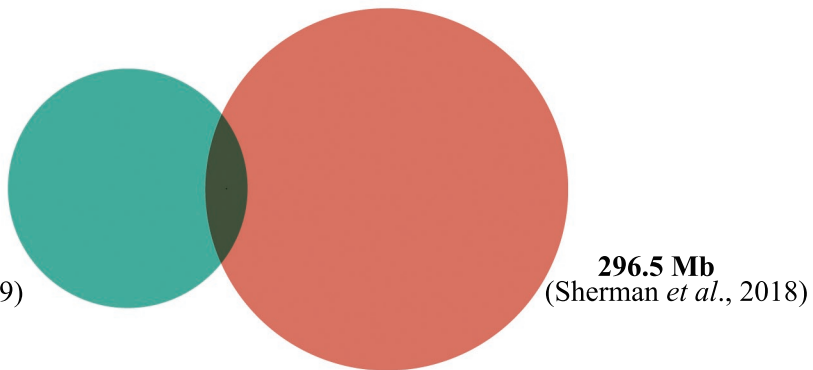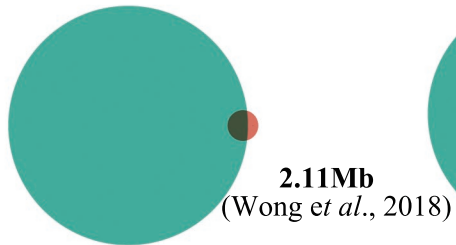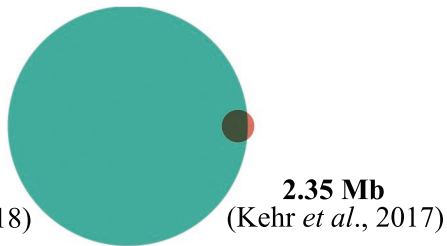

Supplement: Supplementary file 3 — Additional file 3. Comparison of our NRS with four published studies. [file 12864_2019_6107_MOESM3_ESM.pdf]

a

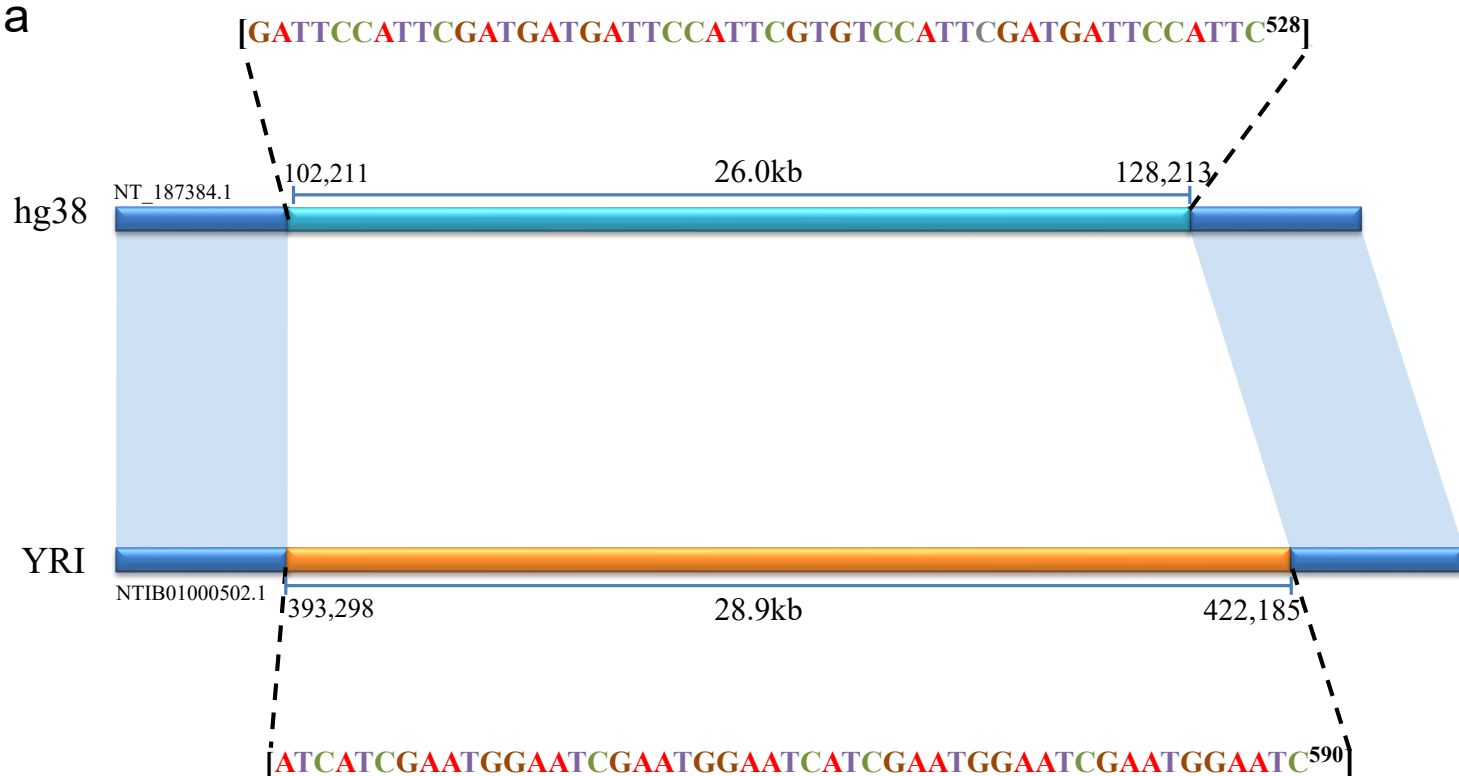

**b**

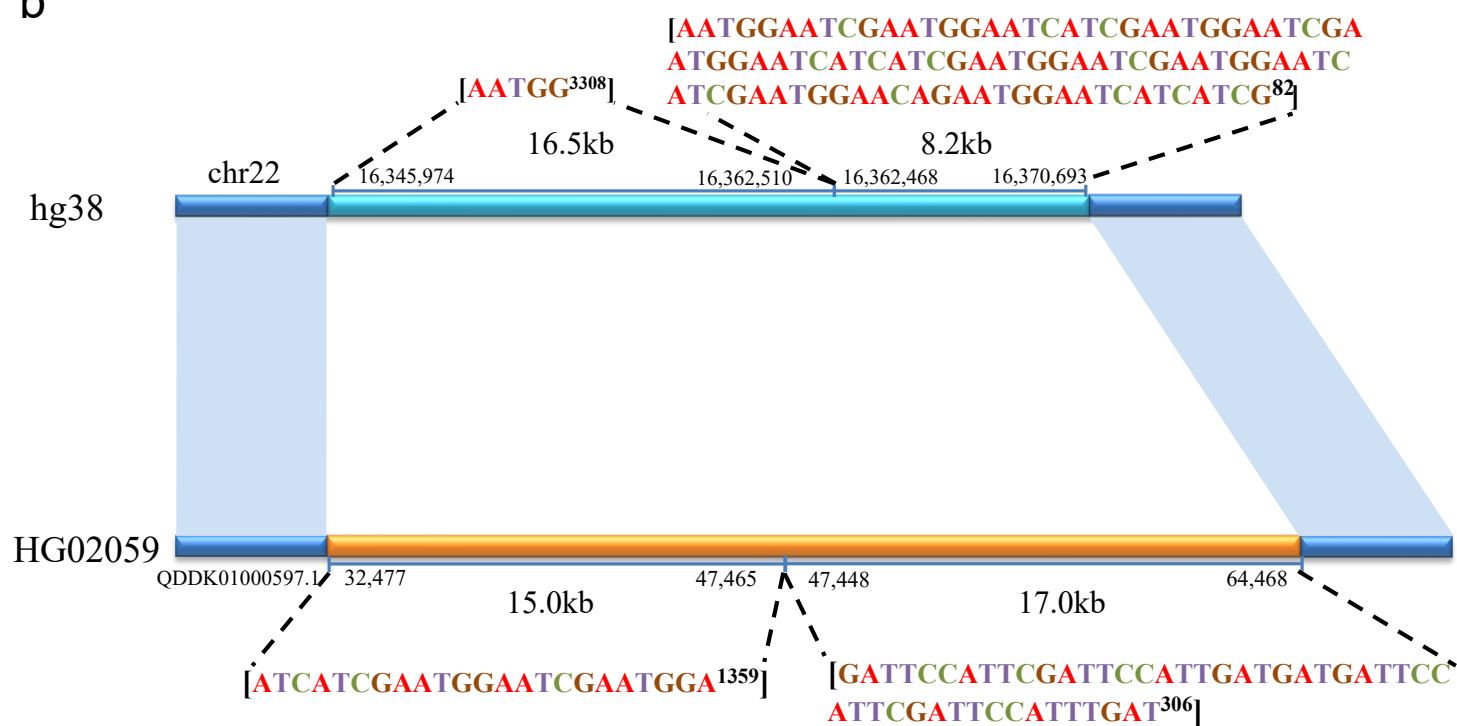

Supplement: Supplementary file 10 — Additional file 10. Two examples of the divergent alleles harboring tandem repeats. [file 12864_2019_6107_MOESM10_ESM.pdf]
