# Supplementary material for: Recovery of non-reference sequences missing from the human reference genome
Source: BMC Genomics. 2019 Oct 16;20:746. doi: 10.1186/s12864-019-6107-1 (PMC6796347; doi:10.1186/s12864-019-6107-1)
Supplement: Supplementary file 7 — Additional file 7. Blastx information of the novel candidate MHC allele. a The two most significant hits show that the novel candidate MHC allele potentially harbors two genes; b Gene description of the two hits; c Sequence alignment of the first hit to known protein; d Sequence alignment of the second hit to known protein. [file 12864_2019_6107_MOESM7_ESM.pdf]

a

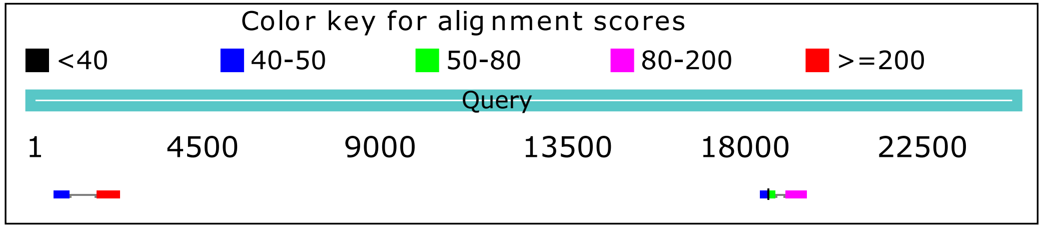

b

Description	Max Score	Total Score	Query Cover	E value	Per. Ident	Accession
PREDICTED: HLA class I histocompatibility antigen, B-39 alpha chain isoform X3 [Chlorocebus sabaeus]	235	284	3%	1.00E-65	74.69%	XP_007971442.1
PREDICTED: uncharacterized protein LOC103247142 [Chlorocebus sabaeus]	159	257	2%	1.00E-40	77.42%	XP_008017372.1

c

Sequence ID: **XP_007971442.1** Length: 385 Number of Matches: 2
Range 1: 203 to 364

Score	Expect	Method	Identities	Positives	Gaps	Frame
235 bits(600)	1e-65()	Compositional matrix adjust.	121/162(75%)	137/162(84%)	0/162(0%)	+2

Features:

```
Query  1625  LFSSDPPKTHMSHQPISDHEATLRFWALVFYPVEITVTQQRDGEDQIQEAELVGTRPAGY  1804
             L  +DP KTH++H P+SDH+A LR WAL +YPVEIT+TQQ+D  +QIQ+AELVGTRP GY
Sbjct  203   LQRADPLKTHVTHHPMSDHKANLRCWALGYYPVEITLTQQQDQNNQIQDAELVGTRPTGY  262

Query  1805  RTFQKWVAVVVSYVKK*RYTCHVQHERLPEPLTLRWGKEMNEGSCVFTGKAGALLEAFSR  1984
             RTFQKW AVVVS  ++ RY CHVQHE LPEPLTLRW KE NEGSC+F+G+AG LLE FS+
Sbjct  263   RTFQKWAAVVVSSGEEQRYACHVQHEGLPEPLTLRWAKEKNEGSCLFSGEAGTLLEPFSQ  322

Query  1985  VRAEA*GSGPLTFCSFLRAIFPAHIPHRGHCYCPGCSWCCGP  2110
             VRAEA GSGPLT  SFLRAIFPAHIPH GH YCPGCSWCCGP
Sbjct  323   VRAEAWGSGPLTVPSFLRAIFPAHIPHCGHHYCPGCSWCCGP  364
```

d

PREDICTED: uncharacterized protein LOC103247142 [Chlorocebus sabaeus]
Sequence ID: **XP_008017372.1** Length: 275 Number of Matches: 3
Range 1: 11 to 165

Score	Expect	Method	Identities	Positives	Gaps	Frame
159 bits(401)	1e-40()	Compositional matrix adjust.	120/155(77%)	123/155(79%)	0/155(0%)	-3

Features:

```
Query  19103  HVTASAWDRSGKGREAALSPHSVSGSALGTADLVSFQ*PSLQALTEPHSGCKLGSSLKGR  18924
              H    AW RSGKGREA LSP SVS SAL  ADL SFQ  SLQ+L  PHSG KLGSSLKGR
Sbjct  11     HRVRLAWGRSGKGREAELSPDSVSRSALVPADLGSFQRSSLQSLMGPHSGSKLGSSLKGR  70

Query  18923  DPERLFPGPLVVSFPAGGGARFHQNRPEADAAF*GWRSSRKRFRILESVGGPWErlrris  18744
              DP RLF G LVV FP+ GGA F+QN PEA AAF G RSSRKRFRILESVGGPWE    IS
Sbjct  71     DPGRLFLGLLVVGFPSDGGAPFYQNGPEAGAAFPGRRSSRKRFRILESVGGPWEGQELIS  130

Query  18743  lltrlrihRGAVssesdsrsrsspdsEFLPRGSAF  18639
               LTRLRIHRGAVSSESDSRS SSPDSEFLPRGSAF
Sbjct  131    QLTRLRIHRGAVSSESDSRSHSSPDSEFLPRGSAF  165
```

Range 2: 171 to 207

Score	Expect	Method	Identities	Positives	Gaps	Frame
50.8 bits(120)	1e-11(2)	Compositional matrix adjust.	28/37(76%)	31/37(83%)	1/37(2%)	-1

Features:

```
Query  18511  QGGVMGDPSRRSELR-ADLKTRAGDQRSPLLSYTSNK  18404
              QGGV GDPS RSELR ADLKTRAG QRS +LSYT ++
Sbjct  171    QGGVTGDPSPRSELRAADLKTRAGAQRSLVLSYTRDR  207
```

Range 3: 200 to 236

Score	Expect	Method	Identities	Positives	Gaps	Frame
47.8 bits(112)	1e-11(2)	Compositional matrix adjust.	23/37(62%)	27/37(72%)	0/37(0%)	-2

Features:

```
Query  18420  VTQATSDCSGPFVFLFRTQQWRLKSARGKSLVCW*VQ  18310
              V   T D +  F+FLFRTQQWRLKSAR +S VCW +Q
Sbjct  200    VLSYTRDRARTFLFLFRTQQWRLKSARRRSPVCWSLQ  236
```
